# Comparative In Vitro Controlled Release Studies on the Chronobiotic Hormone Melatonin from Cyclodextrins-Containing Matrices and Cyclodextrin: Melatonin Complexes

**DOI:** 10.3390/ijms18081641

**Published:** 2017-07-28

**Authors:** Marilena Vlachou, Marianna Papamichael, Angeliki Siamidi, Irene Fragouli, Pandelis A. Afroudakis, Rodanthi Kompogennitaki, Yannis Dotsikas

**Affiliations:** 1 School of Health Sciences, Department of Pharmacy, Section of Pharmaceutical Technology, National and Kapodistrian University of Athens, Panepistimioupoli-Zografou, 15784 Athens, Greece; mpapami1983@gmail.com (M.P.); asiamidi@pharm.uoa.gr (A.S.); esfragouli@gmail.com (I.F.); 2 School of Health Sciences, Department of Pharmacy, Section of Pharmaceutical Chemistry, National and Kapodistrian University of Athens, Panepistimioupoli-Zografou, 15784 Athens, Greece; pafroudakischem@yahoo.gr (P.A.A.); Kompogennitaki@hotmail.com (R.K.); idotsik@pharm.uoa.gr (Y.D.)

**Keywords:** melatonin, cyclodextrins, NMR studies, in vitro controlled release

## Abstract

A series of hydrophilic matrix tablets was prepared and tested with respect to their ability to release the hormone melatonin in a controlled manner, in order to alleviate sleep onset and sleep maintenance dysfunctions. Besides the active ingredient, the tablets were comprised of combinations of the following: HPMC K 15M, low viscosity sodium alginate, microcrystalline cellulose (Avicel PH 102), magnesium stearate, and the cyclodextrins, α-CD, β-CD, γ-CD, HP-β-CD, sulfated β-CD, HP-α-CD and HP-γ-CD, and MLT (guest):CD (host) complexes of the above cyclodextrins, in 1:1 ratio. The controlled release studies were conducted in two aqueous dissolution media at pH 1.2 and 7.4. The stoichiometry of the formed complexes was examined by applying the continuous variation method (Job plot), while the stability constants were calculated by monitoring the spectrophotometric properties of free and CD-encapsulated melatonin (UV-Vis). Host-guest interactions were studied by Nuclear Magnetic Resonance (NMR) spectroscopy. The dissolution data suggest that melatonin is released faster from the MLT:CD complexes than from the rest matrix systems. This enhancement in the dissolution rate and the % release of melatonin from the complexes is due to the increased solubility of the MLT:CD complexes.

## 1. Introduction

The pineal gland neurohormone melatonin (*N*-acetyl-5-methoxytryptamine, MLT) has an important role in the regulation of mammalian circadian rhythms. The secretion of melatonin, which is controlled by a circadian clock in the hypothalamic suprachiasmatic nucleus (SCN), is closely synchronized with the habitual hours of sleep in humans [1]. Recent studies have indicated that increasing serum melatonin concentrations can also trigger the onset of sleep [2]. However, the use of melatonin as a drug is hampered by its short biological half-life and poor bioavailability [3], possibly resulting from extensive first-pass metabolism and variable absorption [4].

During the last fifteen years we have conducted numerous studies in order to decipher the release characteristics of melatonin and its analogues from controlled release matrix tablets [5,6,7,8]. Controlled release versus immediate release has been chosen because melatonin is known to be more clinically useful in initiating and maintaining sleep in elderly insomniacs compared with immediate release or conventional therapy [9].

In this work we have extended our studies on MLT’s in vitro release, by developing new matrix tablets containing cyclodextrins and cyclodextrins:MLT complexes and a combination of other carefully selected excipients. The choice of using cyclodextrins, as melatonin complexing agents, was based on their known ability to increase the aqueous solubility of poorly water soluble drugs and to increase their bioavailability and stability [10]. In addition, cyclodextrins (CD) have been used to reduce gastrointestinal drug irritation, convert liquid drugs into microcrystalline or amorphous powder, and prevent drug–drug and drug–excipient interactions. Previous studies describing complexation of MLT with CDs [11,12] provided some initial data for the current study.

**Figure 1:**
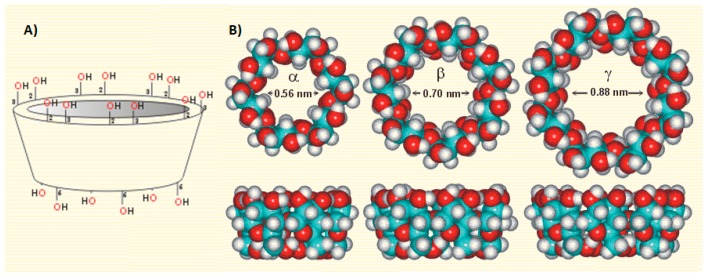
(**Α**) Truncate cone structure of β-CD; (**B**) Top and side view molecular arrangement of α-, β- and γ-CD (http://www1.lsbu.ac.uk/water/cyclodextrin.html).

CDs are a well-known group of water-soluble macromolecules, which form inclusion complexes with a great variety of guest molecules. This property is attributed to their shape, which is like a truncated cone, and to their relatively hydrophobic cavity. There are three common cyclodextrins with 6, 7 or 8 d-glucopyranonsyl residues (α-, β-, and γ-cyclodextrin respectively) linked by α-1,4 glycosidic bonds (Figure 1).

Among these various types of cyclodextrins, β-cyclodextrin is widely used because it is readily available, and its cavity size is suitable for a wide range of guest molecules [13,14]. The inclusion complex of these host–guest systems occurs through various interactions, such as hydrogen bonding, van der Waals intermolecular forces, hydrophobic interactions, and also electrostatic attractions [15], where the described types of bonding would alter the physicochemical and photochemical properties of the guest molecules [16]. Substitution of any of the hydrogen bond-forming hydroxyl groups, even by lipophilic methoxy groups, results in improvement in their aqueous solubility [17]. Water-soluble cyclodextrin derivatives of commercial interest include the hydroxypropyl derivatives of α-CD, β-CD and γ-CD, the methylated β-cyclodextrin (M-β*-*CD), and sulfated β-cyclodextrin (S-β*-*CD). The cavities of CDs have different diameters dependent on the number of glucose units. The side rim depth is the same for all three (about 0.8 nm).

The cyclodextrins used in the present work were: α-CD, β-CD, γ-CD, HP-α-CD, HP-β-CD, HP-γ-CD and S-β-CD as excipients and in melatonin:cyclodextrin complexes in 1:1 ratio. The in vitro controlled release studies were conducted in two dissolution media at pH 1.2 and 7.4.

## 2. Results and Discussion

### 2.1. Estimation of MLT-CD Complexes’ Stoichiometry

Preliminary experiments were conducted in MLT solutions (2 × 10^−5^ M), containing CD (1:10 ratio) in order to estimate the time needed for the stabilization of Δ*A* values (in the absence and presence of the respective CD), due to the MLT encapsulation. Samples were removed from the shaking bath periodically up to 72 h and the absorbance at 278 nm was measured. The absorbance was constantly decreasing, as a result of the complex formation and therefore the absolute value of Δ*A* was increasing. Δ*A* reached its maximum value at 24 h for all CDs tested and then it stabilized. In order to preclude any effect of CD concentration on this time, experiments were repeated with a 1:1 MLT:CD ratio. Again, via similar plots, the same conclusions were reached. Consequently, in the next experiments involving utilization of Δ*A* values, measurements of absorbance were conducted 24 h after MLT’s encapsulation, in a shaking bath at 37 ± 0.5 °C (100 rpm).

The first experiments for the determination of the Δ*A* values involved the estimation of MLT-CD complexes stoichiometries. This information doesn’t actually have a theoretical meaning, since it is connected with a series of assumptions for the determination of stability constant *K*. Based on the Job plot theory (see Section 3.1), plots of Δ*A* (278 nm) vs. *r* for all MLT-CD complexes revealed a Δ*A* maximum at *r* = 0.5. This is indicative for a MLT:CD 1:1 complex, regardless of the CD type. Judging from the MLT structure, this was the most meaningful scenario right from the beginning.

### 2.2. Determination of MLT-CD Complexes Stability Constants

Stability constant *K* is a characteristic value for the intensity of bonds that stabilize the complex. Monitoring of the difference of a physical parameter that can be measured (Δ*A* for absorbance, Δδ for NMR, etc.) one can get a first idea for comparing various CD complexes with the same guest. However, in order to get more valid conclusions, the determination of the complex stability constant *K* is obligatory.

A prerequisite for such study is the estimation of the complex stoichiometry. The fact that in all MLT:CD complexes the stoichiometry is 1:1 simplifies the procedure, since with the suitable assumptions, linear models can be generated (Benesi–Hildebrand equation).

Results for *K* based on Δ*A* values are presented in Table 1, showing excellent linearity (*r*^2^ > 0.99) for the obtained models. One can notice great variation in *K* values and this can be easily attributed to two factors: (a) diameter of the CD cavity and (b) additional groups in the external region of the chemically modified CDs (hydroxypropyl- and S-CD). Focusing on just the native CDs (α-, β-, γ-), it is obvious that the cavity diameter is the critical factor. β-CD is ideal for hosting phenyl/benzyl groups, α-CD is not suitable to host effectively these chromophores and with γ-CD the strength of the bonds is decreased. Usually, the *K* value for a compound bearing an aryl group, encapsulated in β-CD is higher from the *K* in an analogous α-CD and γ-CD complex system. In the case of melatonin’s complexes, the *K* value is even lower. This can be attributed to the structure of MLT, which does not contain a single aryl ring and therefore the fit is less extended. This handicap can be circumvented by replacing the native with a chemically modified CD. The presence of additional polar groups in the external region of CD, such as –OH, can stabilize the complex with additional hydrogen bonds, as in the case of HP-β-CD, resulting in intensification of complex stabilization, as the obtained *K* value of ‎24,092.80 ‎M^−1^ indicates.

### 2.3. Monitoring of MLT Complexes with UV-Vis

**Figure 2:**
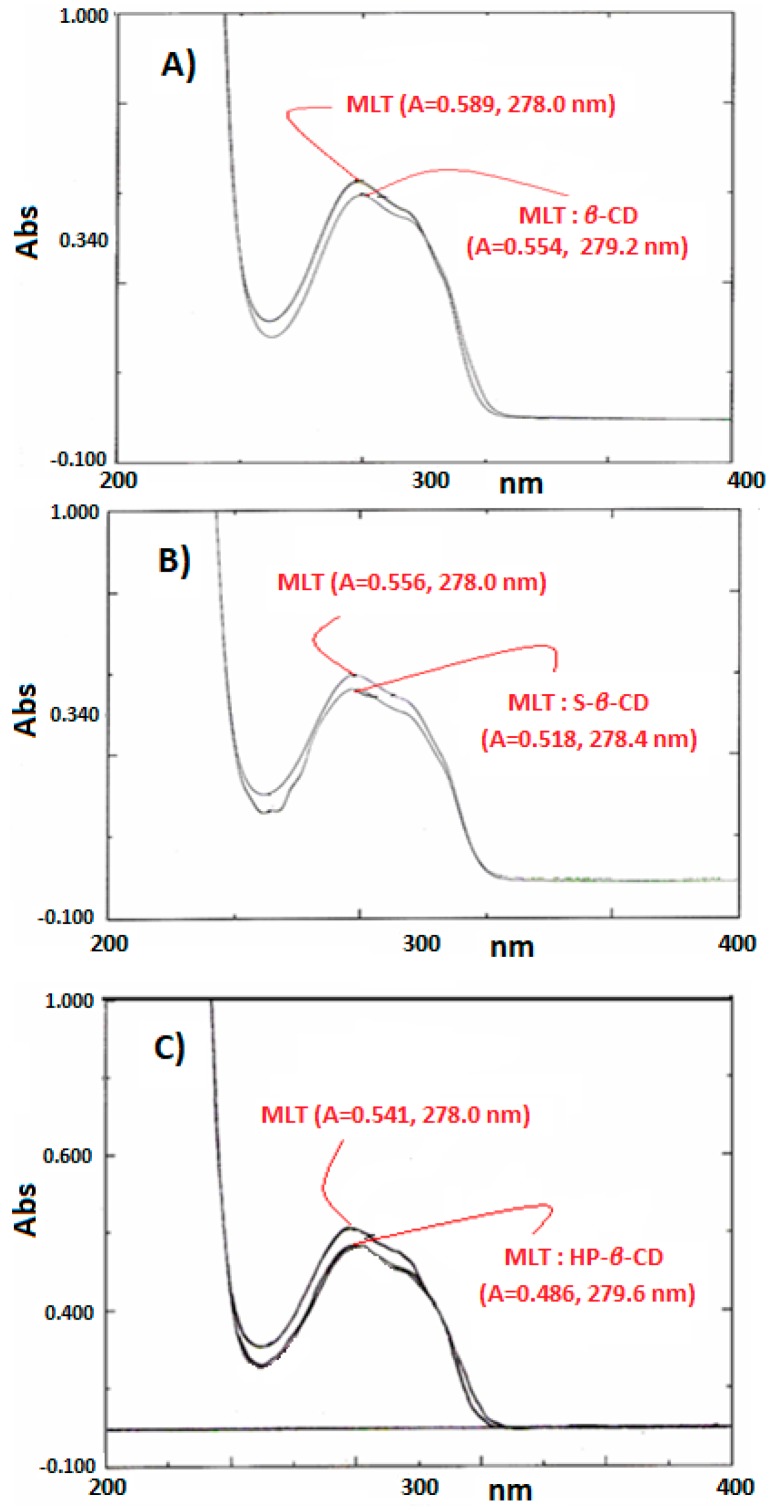
(**A**) UV spectra of MLT:β-CD; (**B**) MLT:S-β-CD; (**C**) MLT:HP-β-CD complexes.

UV-Vis spectrophotometry can provide indications of complex formation by monitoring either the shift of λ_max_ or the difference in absorbance. In the UV-Vis spectra recorded in this work, the λ_max_ values of the MLT:CD complexes showed a slight bathochromic shift with respect to melatonin (Figure 2).

### 2.4. Confirmation of MLT Complex Formation via NMR Spectroscopy

**Figure 3:**
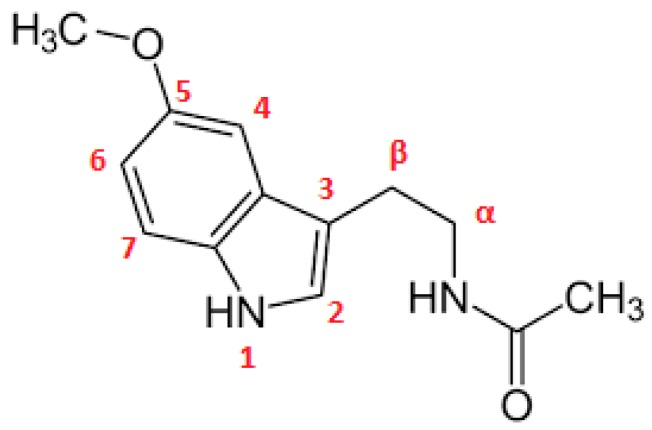
Chemical structure of melatonin (MLT).

**Figure 4:**
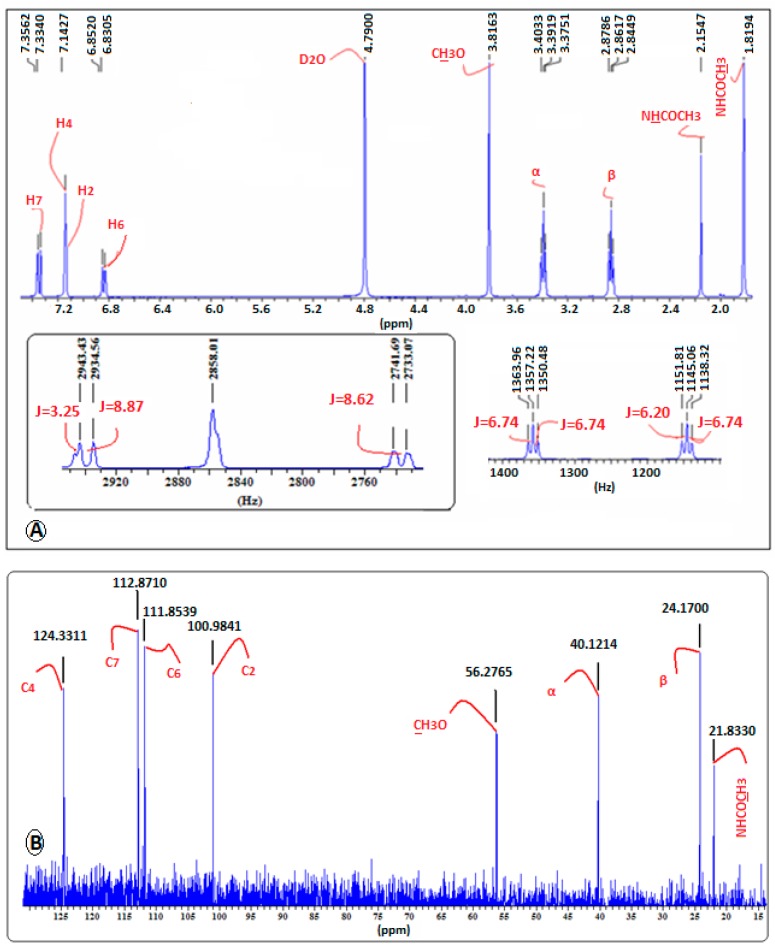
(**A**) ^1^H-NMR; (**B**) ^13^C-NMR spectra of MLT in D_2_O.

**Figure 5:**
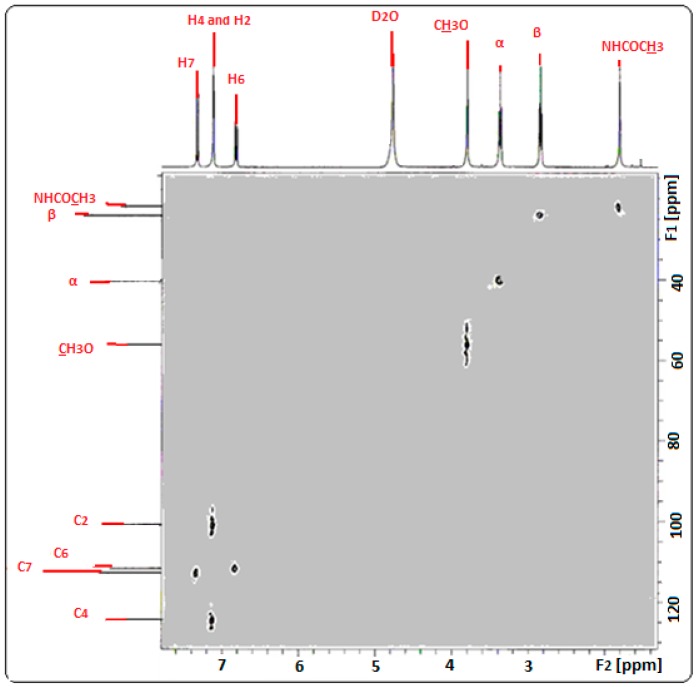
2D NMR spectrum of MLT in D_2_O.

The assignment of the protons of melatonin (Figure 3) was based on its ^1^H-NMR and ^13^C-NMR spectral data (Figure 4) and mainly on the protons–^13^C–NMR correlations observed in its 2D ^1^H–^13^C NMR spectrum (Figure 5).

The effective complex formation of melatonin with the cyclodextrins used was evidenced by the chemical transpositions of most of the aromatic protons of melatonin (0.01–0.07 ppm, mainly downfield shift). This movement to lower magnetic field is probably due to H-bond formation between MLT’s indolic hydrogens and the C3 and C5 hydroxyls of the respective cyclodextrins.

**Figure 6:**
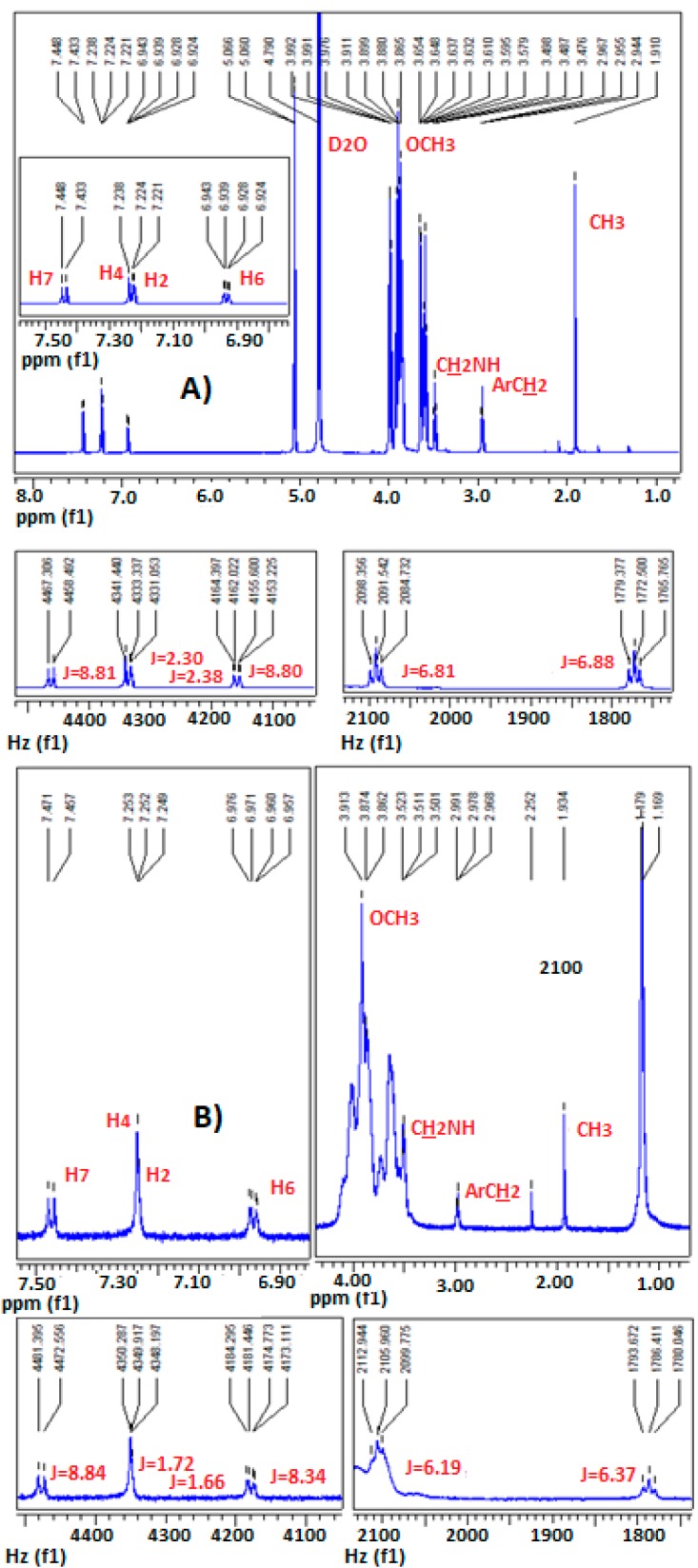
(**A**) ^1^H-NMR spectra of MLT:α-CD; (**B**) MLT:HP-α-CD complex in D_2_O.

In detail, in the ^1^H-NMR spectrum of the MLT:α-CD complex, a downfield shift of the H7 and H4 protons of the MLT indole ring is observed (Figure 6). This is attributed to H-bond formation between these protons and the oxygen atoms of the C3 and C5 OHs of α-CD [18]. Similarly, the H6 proton moves to lower field, but to a lesser extent. Conversely, the H2 proton is shielded in the MLT:α-CD complex, probably due to absence of H-bonding between this proton and the C3 and C5 OHs, located in the inner part of α-CD. It is thus possible that melatonin enters the cavity of the α-CD from the benzene ring site of the indole nucleus. Interestingly, all the aromatic protons of melatonin in the ^1^H-NMR spectrum of the MLT:HP-α-CD complex (Figure 6) show a downfield shift. This implies that melatonin enters the cavity of HP-α-CD, in a different orientation than in α-CD.

**Figure 7:**
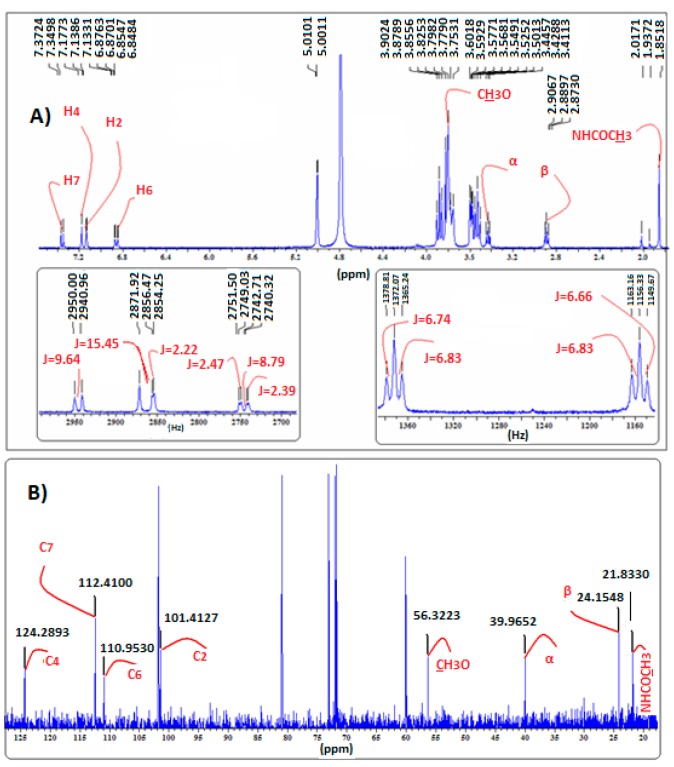
(**A**) ^1^H-NMR; (**B**) ^13^C-NMR spectra of MLT:β-CD complex in D_2_O.

In the case of the MLT:β-CD complex, the ^1^H-NMR spectrum (Figure 7) indicates that all the aromatic protons of melatonin move, due to paramagnetic deshielding, to higher δ values, with proton H4 shifting by 0.03 ppm. Protons H6, H7, and H2 are shifted downfield by 0.02, 0.01, and 0.01 ppm, respectively. These findings are in agreement with the analogous results previously found [18]. Hence, it becomes apparent that MLT’s H2 proton in the MLT:β-CD complex, conversely to the MLT:α-CD complex, forms a H-bond either with the oxygen OH atom of C3 or C5 of β-CD.

**Figure 8:**
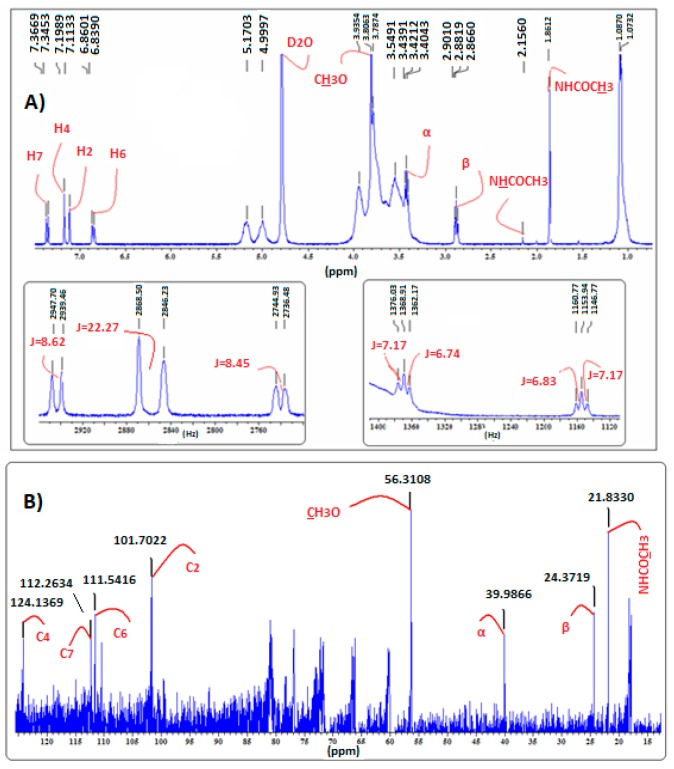
(**A**) ^1^H-NMR; (**B**) ^13^C-NMR (bottom) spectra of MLT:HP-β-CD complex in D_2_O.

**Figure 9:**
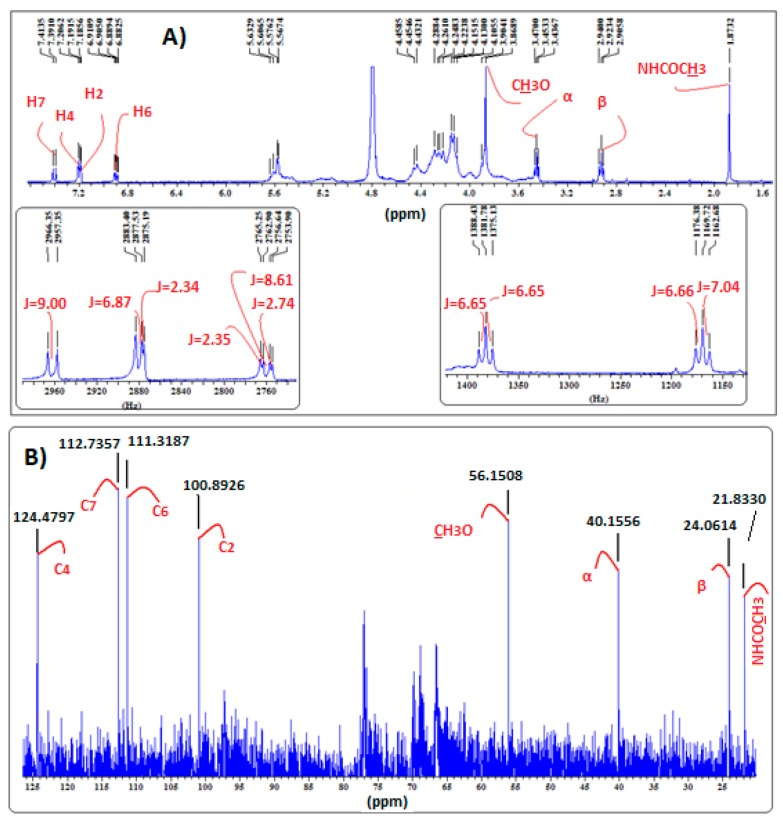
(**A**) ^1^H-NMR; (**B**) ^13^C-NMR spectra of the MLT:S-β-CD complex in D_2_O.

In the ^1^H-NMR spectra of MLT:HP-β-CD (Figure 8), MLT:β-CD (Figure 7), and MLT:S-β-CD (Figure 9) complexes, MLT’s peaks of H4 and H2 appear separately, conversely to their appearance in the ^1^H-NMR spectrum of non-complexed melatonin (Figure 4), where the respective signals almost collapse into one peak. This differentiation in all of the above three complexes is attributed to the inclusion of the indolic part of MLT into the respective CDs cavities. Moreover, the coupling constant (*J*) values were also altered upon complex formation of MLT with these CDs due to intermolecular MLT:CD interactions. The splitting of the H2 (coupling with pyrrolic the NH1 proton) and H6 (meta-interaction with H4) peaks is observed in non-complexed MLT, although they remain as such in the MLT:β-CD and MLT:S-β-CD complexes, they resonate at different δ values due to their intermolecular interactions with the anomeric oxygen atoms in the CDs cavities. Although almost all of the indolic protons of the MLT structure showed in the NMR spectra of the complexes considerable differentiations, the methylene protons of the C3 side chain of melatonin did not seem to be affected by the complexation of MLT with these three CDs. This could suggest that only the indolic part of melatonin enters into the CD cavities, leaving the side chain out.

The ^13^C-NMR spectral data of the MLT:β-CD, MLT:HP-β-CD, and MLT:S-β-CD complexes (Figure 7, Figure 8 and Figure 9, respectively) indicate that the aromatic indole ring carbon atoms C4, C7, and C6, correlating to protons H4, H7, and H6, respectively, resonate at higher δ values than when MLT is non-complexed. Moreover, the C2 carbon, correlating to H2, and the carbon atom of the C5-methoxyl group also appear at higher δ values. These shifts indicate that the aromatic protons of MLT and the Me protons of its C5-methoxy group participate via H-bonds and possibly other weaker forces in bonding interactions within the cavities of these CDs. It is plausible that the oxygen atom of the C5-OMe forms H-bonds with the axial C3 and/or C5 of CDs’ hydrogens, whilst the H4, H7, and H6 protons, the respective carbon atoms of which are shifted downfield by 0.2–0.9 ppm, participate in H-bond formation with the anomeric oxygens of these CDs. Bonding interactions of this type between hydrogen and oxygen and nitrogen atoms of partially water soluble molecules and CDs are known in the literature, and have been attributed to the lowering of the aqueous-hydrophobic interface area, and also to the excretion of water molecules for the CDs’ cavities [19]. These effects lead to a decrease of the Δ*H* value and to a concurrent increase of the Δ*S* in the molecule:CD systems. Moreover, the results of the above studies suggest that the C3 side chain of melatonin, albeit appearing to participate in bonds formation with the OHs of all three of these CDs, might not enter in their cavity, as the Δδ values noticed in this case are within the limits of experimental error.

**Figure 10:**
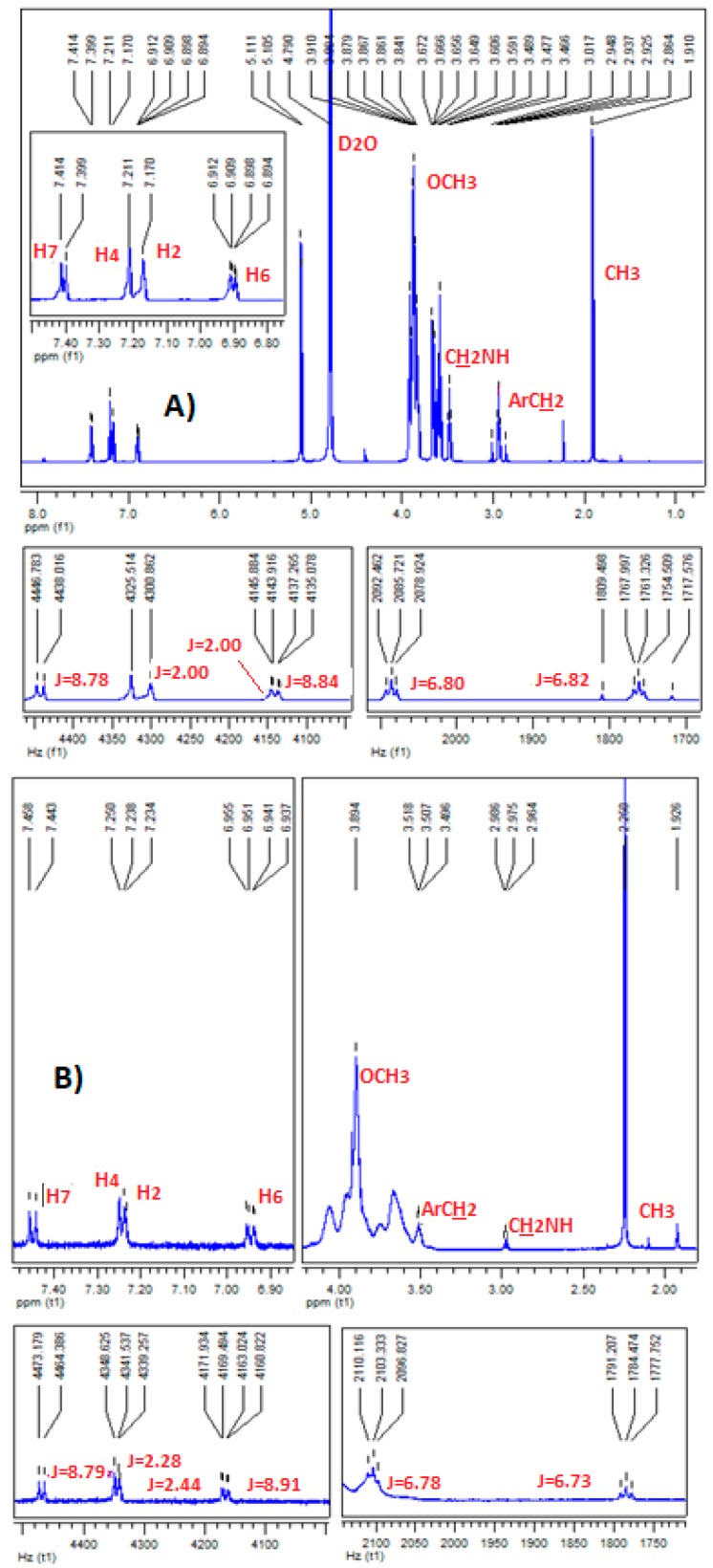
(**A**) ^1^H-NMR spectra of the MLT:γ-CD; (**B**) MLT:HP-γ-CD complexes in D_2_O.

In the case of the MLT:γ-CD complex, no H-bond formation is apparent between the aromatic protons of melatonin and the C3, C5-OHs of γ-CD. However, in the ^1^H-NMR spectrum of the MLT:γ-CD complex, all the protons of the MLT’s indole ring show an upfield shift (Figure 10). This justifies complex formation, not due to the development of H-bonds, but due to weak attractive forces between the molecule of melatonin and γ-CD.

**Figure 11:**
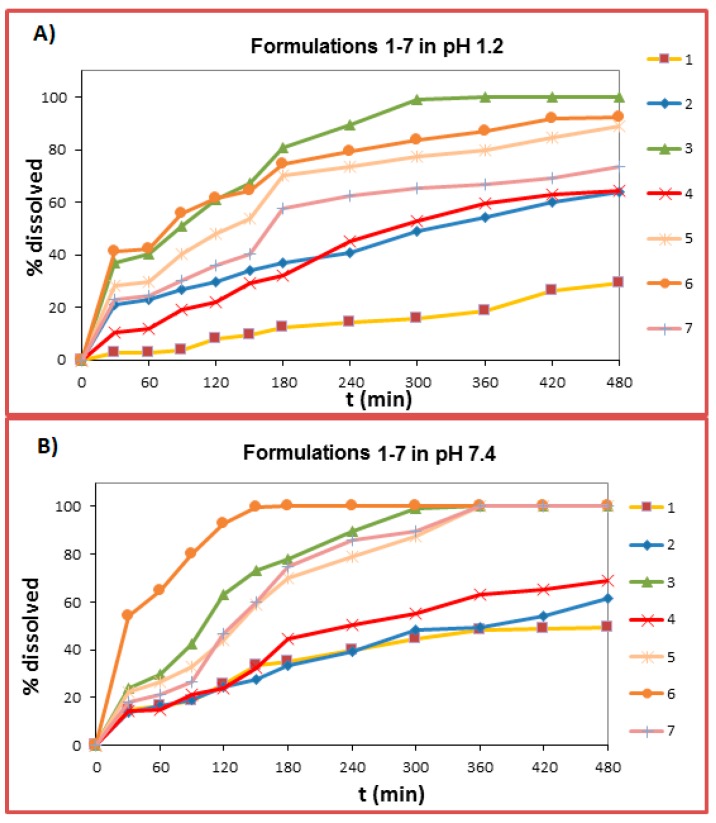
Release curves of melatonin, from tablets containing melatonin/CDs mixtures at (**A**) pH 1.2 and (**B**) pH 7.4 (SD < 2%).

In the ^1^H-NMR spectrum of the MLT:HP-γ-CD complex all the protons of the MLT’s indole ring show a significant downfield shift (Figure 11). Apparently, stronger H-bonds are created between the aromatic protons of melatonin and the C3, C5-OHs of HP-γ-CD than with the respective OHs of native γ-CD. Hence, HP-γ-CD serves as a more suitable host substrate for melatonin than its congeneric γ-CD.

### 2.5. Tablet Formulation and Characterization

Based on the 1:1, MLT:CD ratio, indicated by the estimation studies on MLT-CD complexes’ stoichiometry and the determination of MLT:CD complexes’ stability constants, formulations (F1–F7; CDs present as excipients) and F8–F14 (MLT:CD complexes) were designed and prepared (Table 2).

Formulations F1–F14 presented similar thickness without any change in their weight. Hardness values between 8–10 Kp were considered acceptable. The friability tests were within the limits of USP 39-NF 34 Pharmacopoeia (<1%) in all formulations.

### 2.6. Dissolution Studies

**Figure 12:**
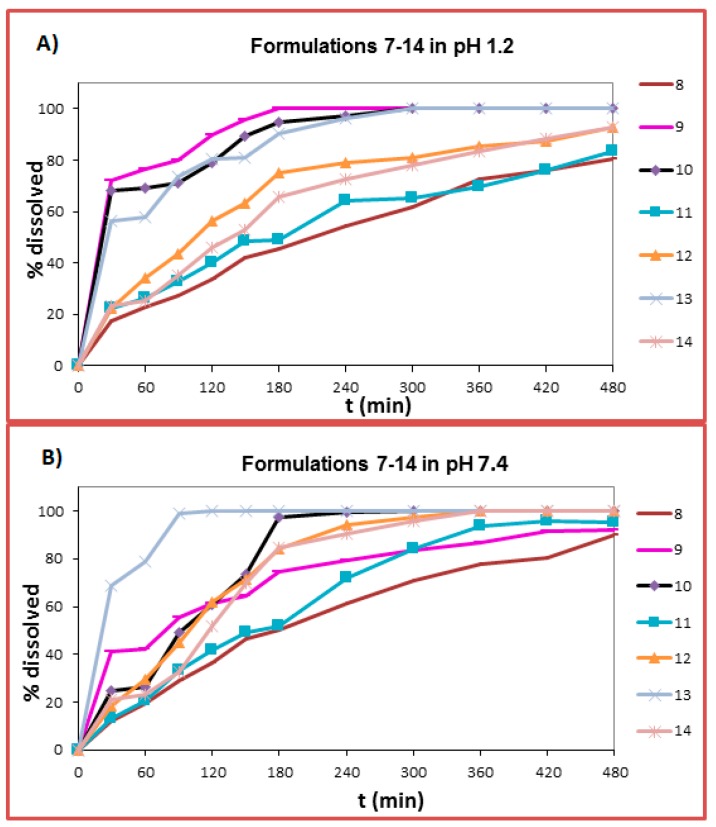
Release curves of melatonin, from tablets containing melatonin:CDs inclusion complexes at (**A**) pH 1.2 and (**B**) pH 7.4 (SD < 2%).

In general, the different rim diameter and cavity size of the CDs used do not seem to drastically affect the ability of melatonin to form MLT:CD complexes [20], possibly because the volume of the molecule of melatonin is quite small (calculated critical volume = 682.5 cm^3^/mol). Nevertheless, for comparison purposes, the dissolution profile of MLT:CDs complexes was probed against that of the respective physical mixtures (not MLT interlaced CDs; CDs present in the tablets as excipients). From the release data presented in Figure 11 and Figure 12, it becomes apparent that from the α-CD, β-CD, γ-CD, HP-α-CD HP-β-CD, HP-γ-CD, and S-β-CD complexes, melatonin is released faster than from the respective physical mixtures, irrespective of the pH of the dissolution medium. The enhancement in the dissolution rate, in the case of the complexes, is due to the increased solubility of the drug:cyclodextrin complex system [21].

When melatonin was interlaced with α-CD, its release reached, at pH 1.2, almost 80% after 8 h and almost 90% after 8 h, at pH 7.4. The calculated *K* value for this complex (1006.9 M^−1^, Table 1) is the lowest from all the calculated *K* values, suggesting a weak host–guest interaction. In the case of MLT:HP-α-CD complex, at pH 1.2, the % release of melatonin was higher, reaching 90% in 8 h, at pH 1.2 and 100% in 6 h at pH 7.4. This is also verified by the greater *K* value obtained for this complex (2857.44 M^−1^, Table 1).

When melatonin was interlaced with HP-β-CD, it developed an initial fast pace of release, which reached, at pH 1.2, almost 100% after 5 h, and 100% after 1.5 h at pH 7.4. This effective release of melatonin from this complex system is corroborated by the NMR spectral data, which suggest a strong host–guest interaction, which is also backed by the calculated *K* value for this complex (24,092.80 M^−1^, Table 1). In the case of the MLT:β-CD complex, the release of melatonin at pH 1.2, followed an analogous pattern. At pH 7.4, the release of melatonin from the MLT:β-CD complex reached almost 90% after 8 h. The release of melatonin from the MLT:S-β-CD complex was initially quite rapid, and it reached 100% after 5 h (pH 1.2), i.e., at approximately the same time from the respective formulation, where S-β-CD served just as an excipient in the tablets. At pH 7.4, the release of melatonin from the MLT:S-β-CD complex, compared to the respective physical mixture, was noticeably improved. Probably, this is due to the anionic group of the particular cyclodextrin, which in the complex system interacts in an electrostatic fashion with the molecule of melatonin at pH 7.4 and not at pH 1.2, where its sulfated group is protonated. This finding is corroborated by the respective NMR spectral data, which suggest an efficient MLT:S-β-CD complexation, which is also verified by the *K* value for this complex (4645.00 M^−1^, Table 1).

When melatonin was interlaced with the γ-CD, its release reached, at pH 1.2, almost 80% after 8 h and almost 90% after 8 h at pH 7.4. The calculated *K* value for this complex was 1638.00 M^−1^ (Table 1). In the case of the MLT:HP-γ-CD complex, the release of melatonin at pH 1.2 was again higher, reaching 90% in 8 h and 100% in 6 h at pH 7.4. This is also verified by the high *K* value for this complex (5431.86 M^−1^, Table 1).

When comparing the dissolution profiles of MLT from complexes with the native α-, β-, and γ-CDs, it is observed that at both pHs, MLT is released faster from its β-CD complex, reaching 100% in 3 h, at pH 1.2, and almost 90% in 8 h, at pH 7.4. This is also backed from the lower Mean Dissolution Time *MDT* value (70.89 at pH 1.2 and 98.60 at pH 7.4, Table 3). On the other hand, from the formulation containing α-CD, the release of melatonin does not reach 100% at both pHs (*MDT* values 157.7 and 162.73 at pH 1.2 and 7.4, respectively, Table 3). From the formulation containing γ-CD, MLT’s release follows an analogous trend at both pHs (*MDT* values 143.12 and 154.41 at pH 1.2 and 7.4, respectively, Table 3).

When comparing the dissolution profiles of MLT from complexes with the substituted HP-α, HP-β and HP-γ-CDs, it is observed that at both pHs, melatonin had a faster release from the HP-β-CD system, reaching 100% in 5 h at pH 1.2, and 100% in 1.5 h at pH 7.4. This is also backed by the lower *MDT* value (65.72 at pH 1.2, and 45.97 at pH 7.4, Table 3). On the other hand, from the formulation containing HP-α-CD and HP-γ-CD the release of melatonin does not reach 100% at pH 1.2 (*MDT* values 114.88 and 113.13 for HP-α-CD and HP-γ-CD respectively, Table 3), At pH 7.4, MLT is fully released (100%) from both formulations in 6 h (*MDT* values 108.34 and 119.22 for HP-α-CD and HP-γ-CD respectively, Table 3).

The mechanism of media penetration to the tablets was found by calculating the *n* values (Table 3). In most cases the *n* values were within 0.45–0.89, indicating anomalous diffusion. It is notable that in the case of F6 (HP-β-CD participates as an excipient), the *n* value was 0.45 at both pHs, indicating a Fickian diffusion mechanism and first order release kinetics.

## 3. Materials and Methods

### 3.1. Determination of MLT–CD Complex Stoichiometry

The stoichiometry of the formed complexes was examined by applying the continuous variation (Job plot) method [22,23], based on the differences in absorbance Δ*Α* of melatonin due to its complexation with the CDs used. More specifically, Δ*Α* values were calculated by measuring the absorbance of melatonin solution in the absence (*A*_0_) and presence (*A*) of the corresponding concentration of the CD. MLT–CD inclusion complexes were prepared in solutions kept in a shaking bath at 37 ± 0.5 °C (100 rpm) with certain molar ratios taking into account the following concentrations ratio, Equation (1):

(1) $$r=\frac{\left[MLT\right]}{\left[MLT\right]+\left[CD\right]}$$

Ratios from *r* = 0 to 1 were utilized, while keeping the denominator constant (10^−5^ M). Samples were filtered (0.45 μm) and their absorbance was measured, having as blank the solution with the respective CD concentration. Solutions with equal MLT concentrations were prepared and their absorbance values were used to estimate the difference Δ*Α*. Then, Δ*Α* was plotted against the denominator of the previous equation and from its maximum, the stoichiometry of the complex was found.

### 3.2. Determination of Stability Constants

For the calculation of the stability constants, the difference in absorbance Δ*Α* was utilized by measuring the absorbance of melatonin solution in the absence (*A*_0_) and presence (*A*) of the increasing concentrations of CD. The following ΜLT:CD ratios were prepared: 1:0.5, 1:1, 1:2, 1:10, 1:20, 1:50. Samples were filtered (0.45 μm) and their absorbance was measured, having as blank the solution with the respective CD concentration. Then, starting from the basic equation of Lambert–Beer law (*A* = ε*bC*), taking into account that free melatonin, free CD and MLT-CD complexes coexist in the solution and having in mind that CDs do not absorb in UV, the following Equation (2) (Benesi–Hildebrand) is generated [22,24] for complexes with 1:1 stoichiometry:

(2) $$\frac{1}{\Delta Α}\;=\;\frac{1}{\Delta \varepsilon_{1:1}\;G_{t}\;{Κ}_{1:1}\;\left[CD\right]}+\;\frac{1}{\Delta \varepsilon_{1:1}\;G_{t}}$$

where, *G*_t_ corresponds to total melatonin concentration, *K*_1:1_ to the stability constant, [CD] to the concentration of CD and Δε_1:1_ to the difference of molar absorptivity of free and complexed melatonin.

By using this equation (1/Δ*Α* vs. 1/[CD]), the calculation of *K*_1:1_ is feasible by utilizing the slope and intercept of the obtained equation.

### 3.3. Preparation of MLT:CD Complexes

The preparation of MLT:CD complexes was performed as follows: The appropriate amounts of the requisite cyclodextrin and solid melatonin were added in a conical flask (25 mL) and distilled water (10 mL) was added. Τhe resulting suspension was stirred with the aid of a magnetic stirrer at room temperature until no solid was observed. The homogeneous solution was then concentrated to dryness, in vacuo (13 mm/Hg) at 45 °C and 100 rpm. At the end, the solid residue obtained was dried further in the oven at 40 °C for 48 h [25].

### 3.4. Instrumentation for Monitoring Host–Guest Interactions

Cyclodextrin:MLT complex formation was monitored by UV-Vis spectrophotometry, at λ_max_ = 278 nm, using a Perkin-Elmer spectrophotometer (Norwalk, CT, USA). Nuclear Magnetic Resonance (^1^H-NMR) spectra were recorded in a Bruker spectrometer (Avance DRX 400, Houston, TX, USA), operating at 400.13 MHz at 25 °C and using D_2_O as solvent. ^13^C-NMR spectra were taken in the same instrument, operating at 100.03 MHz at 25 °C, using D_2_O as solvent and tetramethylsilane (TMS) as internal standard. ^1^H-NMR and ^13^C-NMR chemical shifts were given in parts per million (ppm) relative to that of the solvent signal (DOH: 4.84 ppm), in the case of ^1^H-NMR, and relative to TMS in the case of ^13^C-NMR, with an accuracy of ±0.001 in both cases.

### 3.5. Materials Used for the Preparation of Matrix Tablets

Melatonin was purchased from MP Biomedicasls (Santa Ana, CA, USA). The cyclodextrins α-CD, β-CD, γ-CD, HP-α-CD HP-β-CD, HP-γ-CD and S-β-CD were obtained from Sigma-Aldrich Chemie GmbH (Taufkirchen, Germany). Hydroxypropylmethycellulose (HPMC K 15M), microcrystalline cellulose (Avicel PH 102) and Sodium Alginate (low viscosity) were also obtained from Sigma-Aldrich Chemie GmbH (Taufkirchen, Germany). Magnesium Stearate, which acts as a lubricant, was obtained from Riedel-De Haen (Hannover, Germany).

### 3.6. Preparation of Matrix Tablets

Each matrix tablet was comprised of MLT or MLT-CD complexes and combinations of the excipients as shown in Table 2. The total weight of each tablet, regardless of composition was 200 mg. The flat tablets were produced using a 10 mm diameter die and a hydraulic press (Maassen type, MP 150).

### 3.7. Characterization of Tablets: Tablet Thickness, Hardness, and Friability

The thickness and hardness of the tablets were determined on 10 samples for each batch. The thickness and hardness tests were performed on a Vernier caliper and a hardness tester (Erweka GmbH, type TBH28, Heusenstamm, Germany), respectively. A friabilator (Erweka GmbH, type TA3R) was used for assessing the friability, using 35 samples for each batch. The crushing strength is expressed in Kp and values between 8–10 Kp were considered acceptable. The % friability is reported in terms of weight loss and has been calculated as percentage of the initial weight, according to Pharmacopeia specifications. An initial mass (*W*_0_) of 35 tablets without dust was weighed and put into a drum rotating at a rate of 25 rpm for 4 min. Then, the tablets were carefully dedusted and weighed again (*W*_R_). The % friability of the tablets was computed using the following Equation (3):

(3) $$Friability=\;\frac{W_{0}\;-\;W_{R}}{W_{0}}\;\times 100$$

### 3.8. In Vitro Dissolution Studies

The dissolution experiments involved the tablet formulations F1–14. The tablets were stirred at 50 rpm in a USP XXIII dissolution apparatus II (Pharma Test Apparatebau AG, Hainburg, Germany) for 8 h containing 500 mL of either gastric (pH 1.2) or intestinal fluid (pH 7.4) at 37 ± 0.5 °C. Samples (5 mL) were withdrawn at predetermined time intervals, filtered, and analyzed at λ_max_ = 278 nm, using a Perkin–Elmer UV spectrophotometer (Norwalk, CT, USA). All experiments were carried out in triplicate.

In order to compare the dissolution profiles, graphs of % drug release vs. time were constructed (Figure 11 and Figure 12) and the Dissolution Efficiency (*D.E.*) (%) value was estimated. According to Khan (1975), *D.E.* (%) is a parameter useful for the evaluation of dissolution in vitro and is calculated according to Equation (4):

(4) $$D.E.\left(\%\right)=\frac{\int_{t_{1}}^{t_{2}}y\times dt}{y_{100}\left(t_{2}-t_{1}\right)}\times 100\%$$

where, *y* is the percentage of dissolved product and *D.E.* the area under the dissolution curve between time points *t*_1_ and *t*_2_, expressed as a percentage of the curve at maximum dissolution, *y*_100_ over the same time period. When a relationship between dissolution and another variable is sought, it is considered more realistic to use *D.E.* (%), which takes into account the dissolution profile as a whole [26].

Also, the *t*_20%_, *t*_50%_, *t*_90%_, as well as the Mean Dissolution Time (*MDT*) values were estimated. The *t*_20%_, *t*_50%_ and *t*_90%_ values refer to the time where the 20%, 50% and 90% of the active substance is released. *MDT* is the value used to characterize the drug release rate from a dosage form and the following Equation (5) is used to derive an estimate of *MDT* from experimental dissolution data [27]:

(5) $$MDT=\frac{ABC}{W_{\infty }}$$

where, *W*_∞_ is the maximal amount of the drug substance that is dissolved and ABC is the area between the drug dissolution curve and its asymptote.

The in vitro release data were fitted to the Korsmeyer–Peppas Equation (6):

(6) $$\frac{M_{t}}{M_{\infty }}=Kt^{n}$$

where, *M*_t_ and *M*_∞_ express the absolute cumulative amount of drug released at time t and infinite time, respectively, *k* is the release rate constant and *n* is the diffusion coefficient. This equation is only valid for the first 60% of the fractional release [28]. The values assumed by the *n* exponent represent either Fickian or anomalous (non-Fickian) release kinetics. For the case of cylindrical tablets, in particular, *n* ≤ 0.45 corresponds to a Fickian diffusion release (case I diffusional), 0.45 < *n* < 0.89 to an anomalous transport, and *n* = 0.89 to zero-order (case II) release kinetics.

## 4. Conclusions

We have shown that melatonin:cyclodextrin inclusion systems enhance the hormone’s dissolution rate and % release in a controlled manner, which is affected by the nature of the cyclodextrin used. Currently, we probe the stoichiometry and complexation profiles of other MLT:CD inclusion complexes with respect to their stability and dissolution properties.

## Figures and Tables

**Table 1:** Stability constants (*K*) for MLT complexes, slope, intercept, and coefficient of determination values (*r*^2^) from Benesi–Hildebrand equations

Complex	Slope	Intercept	*r*^2^	*K* (M^−1^)
MLT and α-CD	23.30 × 10^−3^‎	23.623	0.993	1006.90
MLT and β-CD	‎15.00 × 10^−3^‎	‎27.716‎	0.995	1847.70
MLT and S-β-CD	‎‎8.00 × 10^−3^‎	‎37.160‎	0.990	4645.00
MLT and γ-CD	22.70 × 10^−3^‎	37.185	0.997	1638.00
MLT and HP-α-CD	20.70 × 10^−3^‎	59.149	0.991	2857.44
MLT and HP-β-CD	57.98 × 10^−5^‎	‎13.969‎	0.995	24,092.80
MLT and HP-γ-CD	9.70 × 10^−3^‎	52.689	0.991	5431.86

**Table 2:** Melatonin tablet formulations (quantities in mg).

	1	2	3	4	5	6	7	8	9	10	11	12	13	14
MLT	2	2	2	2	2	2	2	-	-	-	-	-	-	-
α-CD	8.8	-	-	-	-	-	-	-	-	-	-	-	-	-
β-CD	-	10.2	-	-	-	-	-	-	-	-	-	-	-	-
S-β-CD	-	-	22.5	-	-	-	-	-	-	-	-	-	-	-
γ-CD	-	-	-	11.7	-	-	-	-	-	-	-	-	-	-
HP-α-CD	-	-	-	-	10.6	-	-	-	-	-	-	-	-	-
HP-β-CD	-	-	-	-	-	13.9	-	-	-	-	-	-	-	-
HP-γ-CD	-	-	-	-	-	-	14.3	-	-	-	-	-	-	-
MLT:α-CD	-	-	-	-	-	-	-	10.8	-	-	-	-	-	-
MLT:β-CD	-	-	-	-	-	-	-	-	12.2	-	-	-	-	-
MLT:S-β-CD	-	-	-	-	-	-	-	-	-	24.5	-	-	-	-
MLT:γ-CD	-	-	-	-	-	-	-	-	-	-	13.7	-	-	-
MLT:HP-α-CD	-	-	-	-	-	-	-	-	-	-	-	12.6	-	-
MLT:HP-β-CD	-	-	-	-	-	-	-	-	-	-	-	-	15.9	-
MLT:HP-γ-CD	-	-	-	-	-	-	-	-	-	-	-	-	-	16.3
HPMC K 15M	30	30	30	30	30	30	30	30	30	30	30	30	30	30
Avicel PH 102	127.2	125.8	113.5	124.3	125.4	122.1	121.7	127.2	125.8	113.5	124.3	125.4	122.1	121.7
Sodium Alginate	30	30	30	30	30	30	30	30	30	30	30	30	30	30
Mg.Stearate	2	2	2	2	2	2	2	2	2	2	2	2	2	2
Total	200	200	200	200	200	200	200	200	200	200	200	200	200	200

**Table 3:** *n*, *t*_20%_, *t*_50%_, *t*_90%_, *MDT* and Dissolution Efficiency % (*D.E.* %) values for all formulations, at pH 1.2 and 7.4.

	pH 1.2						pH 7.4					
	*n*	*t*_20%_	*t*_50%_	*t*_90%_	*MDT*	*D.E.* (%)	*n*	*t_20%_*	*t_50%_*	*t_90%_*	*MDT*	*D.E.* (%)
**F1**	1.03	370	**^b^**	**^b^**	208.93	15.76	0.49	95	**^b^**	**^b^**	121.83	38.41
**F2**	0.49	29	315	**^b^**	149.42	66.00	0.62	95	165	**^b^**	163.96	61.70
**F3**	0.45	16	90	240	103.25	69.70	0.84	23	99	240	109.50	62.30
**F4**	0.85	95	250	**^b^**	167.73	44.13	0.77	82	240	**^b^**	157.28	48.25
**F5**	0.47	22	130	**^b^**	121.10	68.67	0.70	24	131	312	139.56	75.43
**F6**	0.45	15	78	400	92.16	78.60	0.45	10	26	110	77.58	63.00
**F7**	0.64	25	168	**^b^**	124.23	56.50	1.05	49	126	302	143.13	78.26
**F8**	0.62	45	210	**^b^**	157.71	55.79	0.78	60	180	480	162.73	60.84
**F9**	^a^	9	22	120	70.89	70.90	^a^	15	75	400	98.60	46.50
**F10**	0.52	9	23	150	55.90	59.38	0.77	23	92	170	95.99	69.40
**F11**	^a^	25	183	**^b^**	143.12	79.60	0.82	59	155	335	154.41	58.60
**F12**	0.68	25	105	450	114.88	73.24	0.95	32	98	115	108.34	85.57
**F13**	^a^	11	27	80	65.72	76.80	^a^	8	22	85	45.97	54.90
**F14**	0.62	25	138	180	113.13	68.81	0.81	28	116	230	119.22	91.97

**^a^**: Values could not be calculated as the dissolution rate was too fast; **^b^**: values could not be calculated as the dissolution pace was too slow.
